# A Case of Pneumomediastinum and Pneumorrhachis in a Patient With Multiple Risk Factors

**DOI:** 10.7759/cureus.69536

**Published:** 2024-09-16

**Authors:** Toyin Ingram, Ishani Kapoor, Yasmine Azzi, Moses O Evbuomwan, Chika Okafor

**Affiliations:** 1 Internal Medicine, Cape Fear Valley Health, Fayetteville, USA; 2 Internal Medicine, Drexel University College of Medicine, Philadelphia, USA; 3 Radiation Oncology, University of Iowa Hospitals and Clinics, Iowa City, USA

**Keywords:** asymptomatic pneumomediastinum, complications, gastrograffin, mssa bacteremia, pneumorrhachis, preventing complications, rhabdomyolyisis

## Abstract

Pneumomediastinum is the presence of gas (usually air) in the mediastinum, which is rare and typically benign. Pneumomediastinum is classified into primary and secondary based on etiology. Its pathophysiology is due to high intra-alveolar pressures causing alveolar rupture, which releases air that travels along bronchoalveolar sheaths into the mediastinum. Pneumomediastinum may also be concurrently seen with pneumorrhachis defined as air in the spinal canal, although this finding is rare. Here, we present the case of a 27-year-old male with a past medical history of polysubstance use and a one-week history of nausea, vomiting, and chest pain who presented with frostbite and was found to have pneumomediastinum and pneumorrhachis.

## Introduction

Pneumomediastinum is a rare and typically self-limited condition characterized by free air in the mediastinum [1]. It has an estimated prevalence of 0.002% and is most often seen in tall, thin, young males [2]. The Macklin phenomenon explains its pathophysiology. It is thought to be caused by an inciting event, such as physical trauma, causing increased intra-alveolar pressures, which leads to alveolar rupture and air escaping from the lungs, airways, or bowel into the chest cavity [3]. The extravasated air can dissect into neighboring cervical subcutaneous tissues, epidural space, pericardium, or the peritoneal cavity [2]. Typical presenting symptoms include sudden-onset severe, central, or retrosternal chest pain with radiation to the back or neck, dyspnea, cough, and subcutaneous emphysema of the face, neck, and chest [4]. Diagnosis is typically made using a chest X-ray or a computed tomography (CT) scan of the thorax if chest X-ray findings are inconclusive. A particularly telling physical examination finding is Hamman’s sign, a precordial crepitation that is audible and synchronous to the heartbeat [5]. There are two classifications of pneumomediastinum, namely, primary spontaneous pneumomediastinum (PSPM) and secondary pneumomediastinum (SPM). PSPM is associated with risk factors such as tobacco and recreational drug use, specifically, cocaine, marijuana, and methamphetamine use. SPM is associated with respiratory causes (asthma, chronic obstructive pulmonary disease, bronchiectasis, interstitial lung disease, lung cancer, foreign body, excessive vomiting, excessive coughing, childbirth, strenuous physical activity), iatrogenic causes (endoscopy, intubation), or trauma (blunt or penetrating injuries) [1]. Treatment for pneumomediastinum is usually conservative, as the condition is self-limiting as mediastinal tissue slowly reabsorbs the air [1]. Pneumomediastinum may also be concurrently seen with pneumorrhachis defined as air in the spinal canal [6]. Pneumorrhachis was first reported in 1977 by Gordon et al. [7] and was termed pneumorrhachis in 1987 [8]. One suggested explanation for this concurrent finding is that there is no fascia between the posterior mediastinum and retropharyngeal and epidural space, creating a way for air to diffuse into the lower resistance epidural space [6]. Concurrent presentation of pneumomediastinum with pneumorrhachis is rare. Prompt diagnosis is crucial to provide appropriate treatment. Here, we present the case of a 27-year-old male with a medical history significant for polysubstance abuse. He presented to the emergency room with a one-week history of nausea, vomiting, and chest pain. Physical examination revealed nares, fingers, and toes frostbite. He was diagnosed with pneumomediastinum concurrently with pneumorrhachis.

## Case presentation

A 27-year-old male with a past medical history of heroin, fentanyl, cocaine, and marijuana abuse and cannabinoid hyperemesis syndrome who worked as a hunt master was brought to the emergency department (ED) by Emergency Medical Services (EMS) after being found unconscious outside in 16°F weather. According to the family, the patient lived in a shed outside the family home and had been ill for the last two weeks. On arrival at the ED, he had regained consciousness and was awake, alert, and oriented. However, he was unable to recollect what had happened nor explain why he was outside in such temperatures improperly dressed. He was monitored in the ED and eventually was able to recall that he had taken a pill from a friend, which he thought was Percocet. He explained that after ingestion of the pill he had lost electricity supply and had gone outside to attempt to fix it and that was when he syncopized. He was later found by his uncle who made the call to EMS.

Vital signs were significant for a blood pressure of 160/67 mmHg, a pulse rate of 95 beats per minute, a respiratory rate of 20 breaths per minute, and a temperature of 36.7°C (98°F). He was noted to be saturating 98% on room air. He was ill appearing with frostbite injuries to his nose, multiple bruises on his arms and legs, and abrasions with healing scars in various stages of healing throughout his body. A sacral ulcer was also noted. Crepitus was noted over the left side of the chest. Capillary refill was 2-3 seconds. He endorsed a decrease in appetite over the last two weeks, intractable vomiting for two days, nausea, abdominal pain, sore throat, chills, fatigue, sinus pressure, congestion, and sores in his mouth. He also endorsed shortness of breath and chest tightness with palpitations, arthralgias and myalgias. He denied physical abuse but endorsed multiple falls during a hunting competition. He also denied any illicit drug use. His complete blood count is presented in Table 1 and a comprehensive metabolic panel is shown in Table 2.

**Table 1:** Complete blood count results.

Complete blood count	Reference range	Patient’s lab values
White blood cell count	4.5–12.5 × 10^3^/µL	11.4 × 10^3^/µL
Red blood cell count	4.70–6.10 × 10^6^/µL	4.06 × 10^6^/µL
Hemoglobin	13.5–18.0 g/dL	12.3 g/dL
Hematocrit	40.5–54.0 %	36.6%
Mean corpuscular volume	80.0–95.0 fL	90.2 fL
Mean corpuscular hemoglobin concentration	31.0–36.0 g/dL	33.6 g/dL
Platelets	150–450 × 10^3^/µL	197 × 10^3^/µL

**Table 2:** Comprehensive metabolic panel results.

Comprehensive metabolic panel	Reference ranges	Patient’s lab values
Sodium	136–145 mmol/L	128 mmol/L
Potassium	3.4–4.9 mmol/L	2.5 mmol/L
Chloride	98–107 mmol/L	81 mmol/L
Carbon dioxide	21–32 mmol/L	36 mmol/L
Anion gap	1–11 mmol/L	11 mmol/L
Blood urea nitrogen	7–25 mg/dL	34 mg/dL
Creatinine	0.60–1.30 mg/dL	1.12 mg/dL
Estimated glomerular filtration rate	>60.0 mL/minute/1.73m^2^	>60.0 mL/minute/1.73m^2^
Random glucose	74–109 mg/dL	109 mg/dL
Calcium	8.6–10.2 mg/dL	7.4 mg/dL
Alkaline phosphatase	30–105 U/L	43 U/L
Albumin	3.5–5.7 g/dL	3.5 g/dL
Total protein	6.4–8.9 g/dL	5.9 g/ dL
Aspartate aminotransferase	13–39 U/L	42 U/L
Alanine transaminase	7–52 U/L	57 U/L
Total bilirubin	0.3–0.1 mg/dL	0.8 mg/dL
Lactate	0.5–2.0 mmol/L	0.9 mmol/L
Magnesium	1.9–2.7 mg/dL	1.7 mg/dL
Glucose	74–106 mg/dL	101 mg/dL
Creatine kinase	39–308 U/L	1.319 U/L

**Figure 1:**
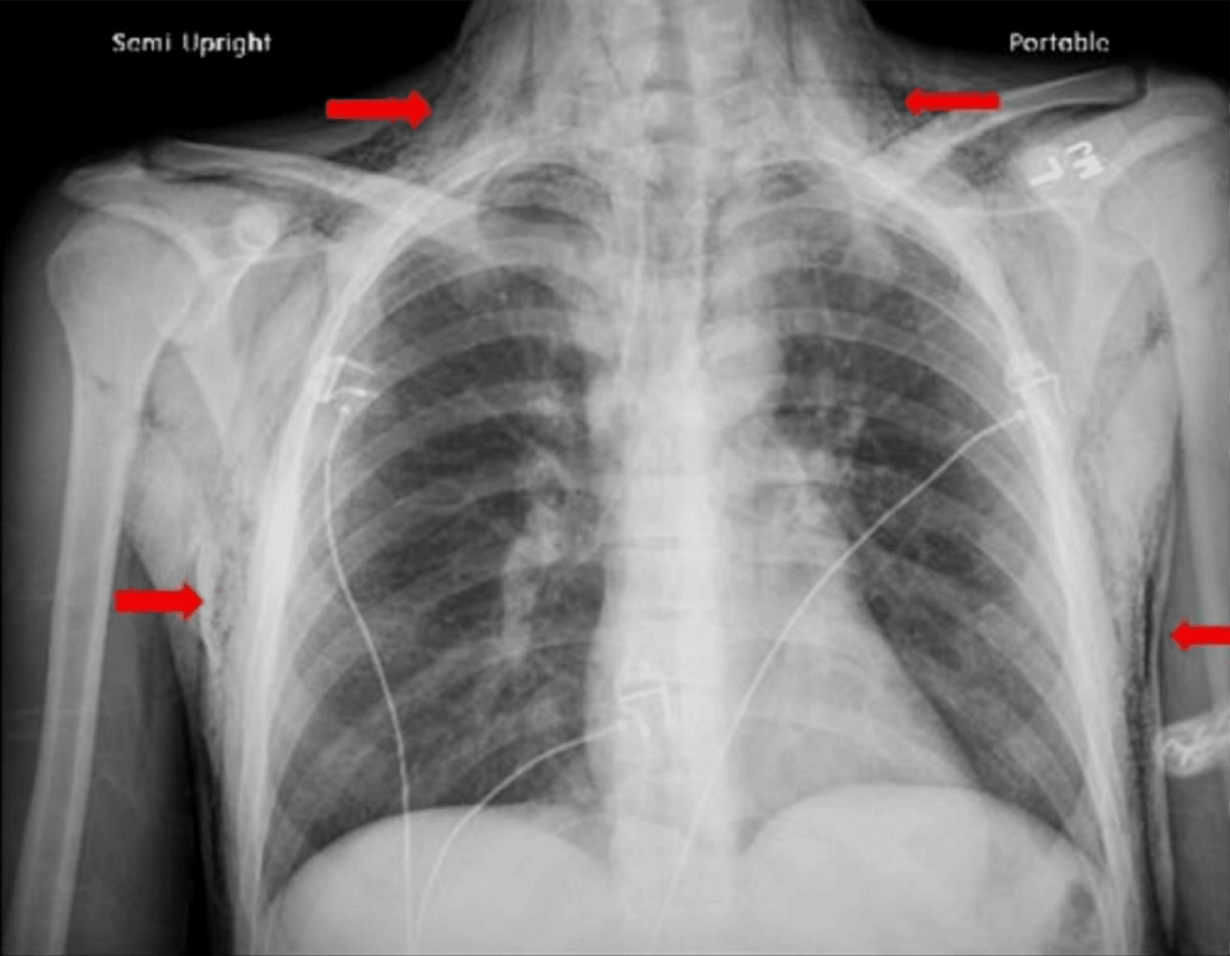
Chest X-ray (red arrows) showing air outlining the mediastinum and extending superiorly along the trachea into the neck.

The patient’s urine drug screen came back positive for fentanyl and opiates. His chest X-ray revealed normal heart size (Figure 1), diffuse soft tissue emphysema, suspected pneumomediastinum, and no focal air space opacities. There were no radiographically apparent pneumothoraxes. Moreover, no discrete acute osseous abnormality was identified.

**Figure 2:**
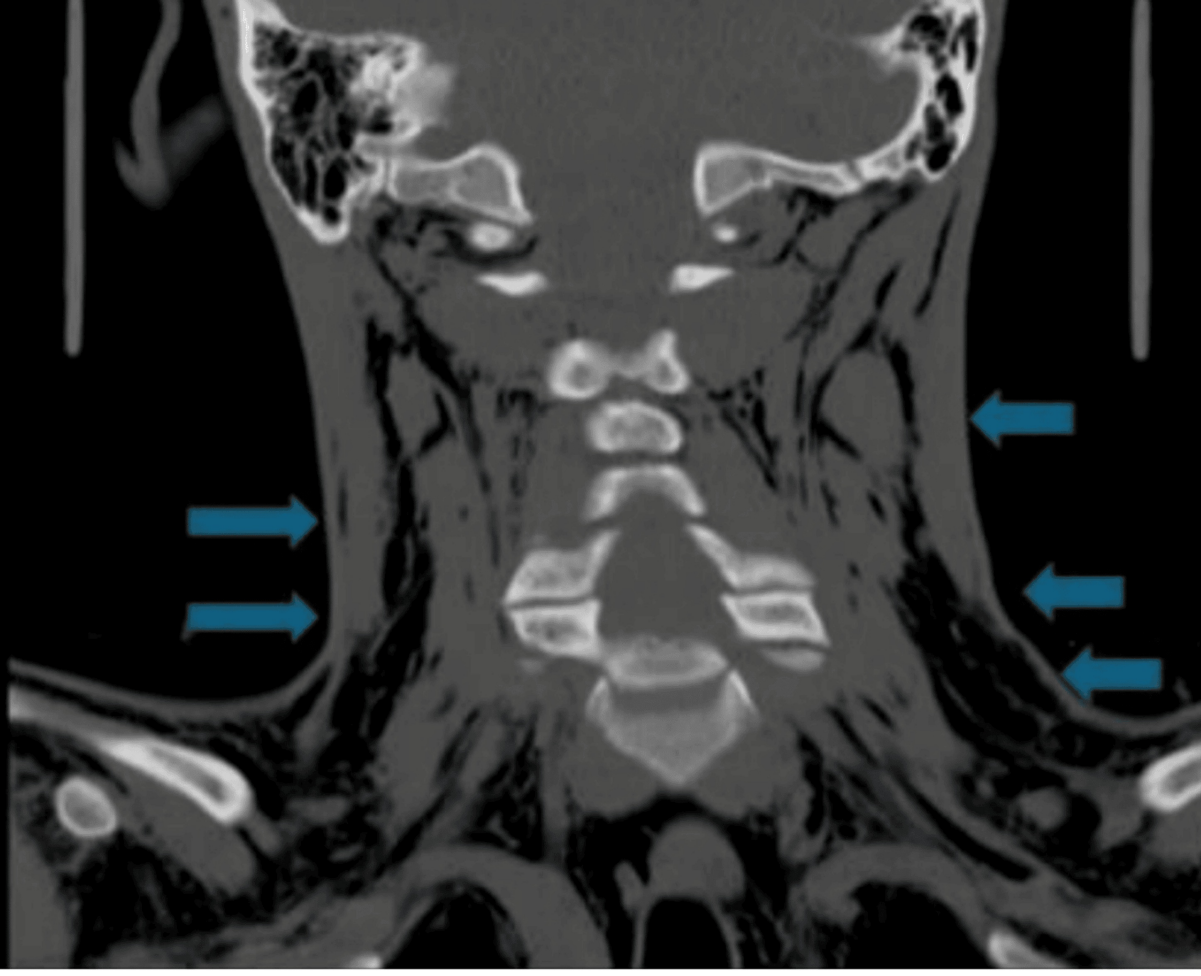
Computed tomography scan (coronal view) of the cervical spine without contrast showing extensive emphysema throughout the soft tissue planes of the upper chest and neck (blue arrows).

CT of the cervical spine without contrast showed no cervical spine fracture (Figure 2). There was extensive emphysema throughout the soft tissue planes of the upper chest and neck. There was pneumomediastinum as well but no pneumothorax.

**Figure 3:**
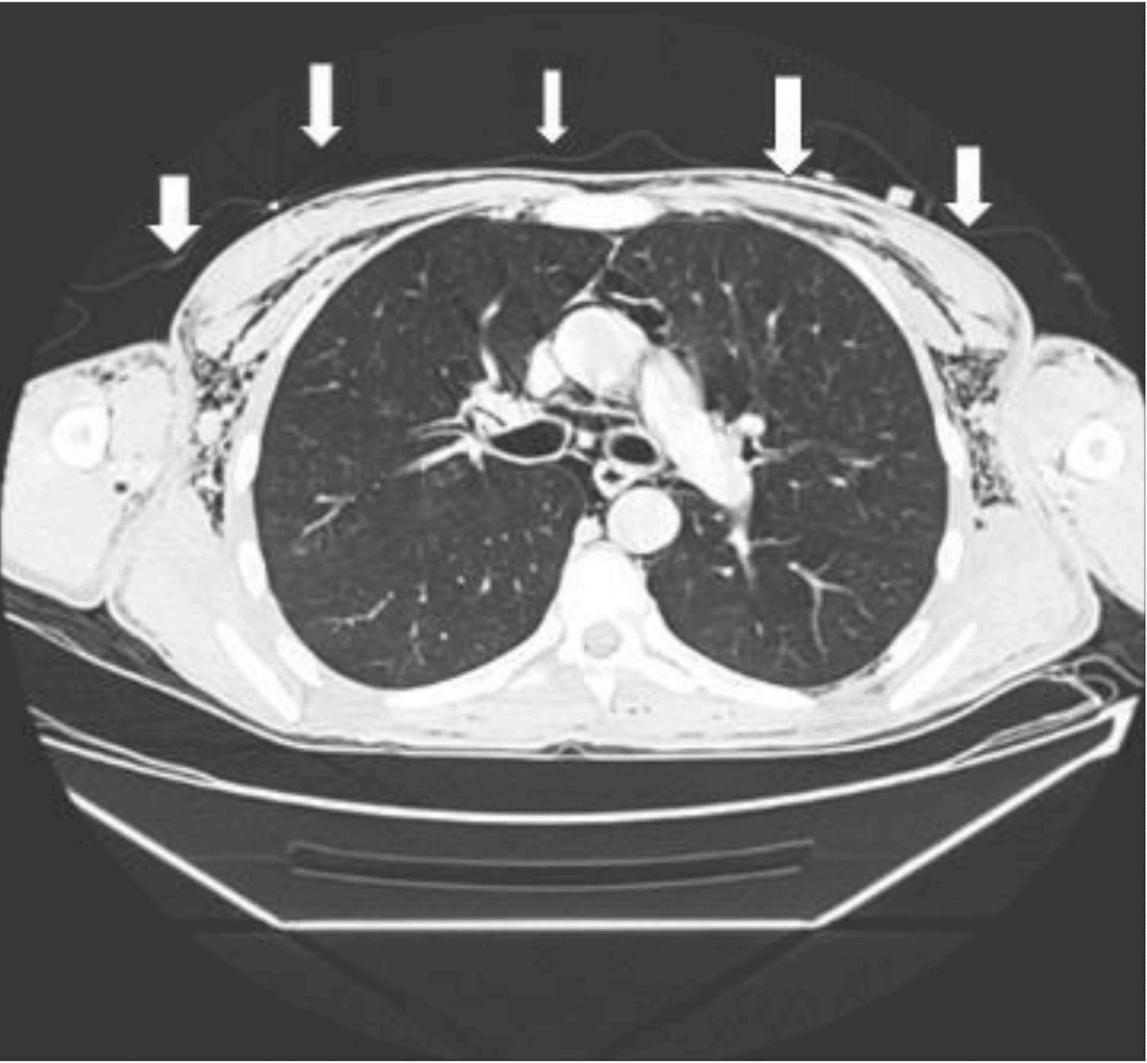
Computed tomography combo body with intravenous contrast scan (axial view) showing extensive pneumomediastinum (white arrows).

**Figure 4:**
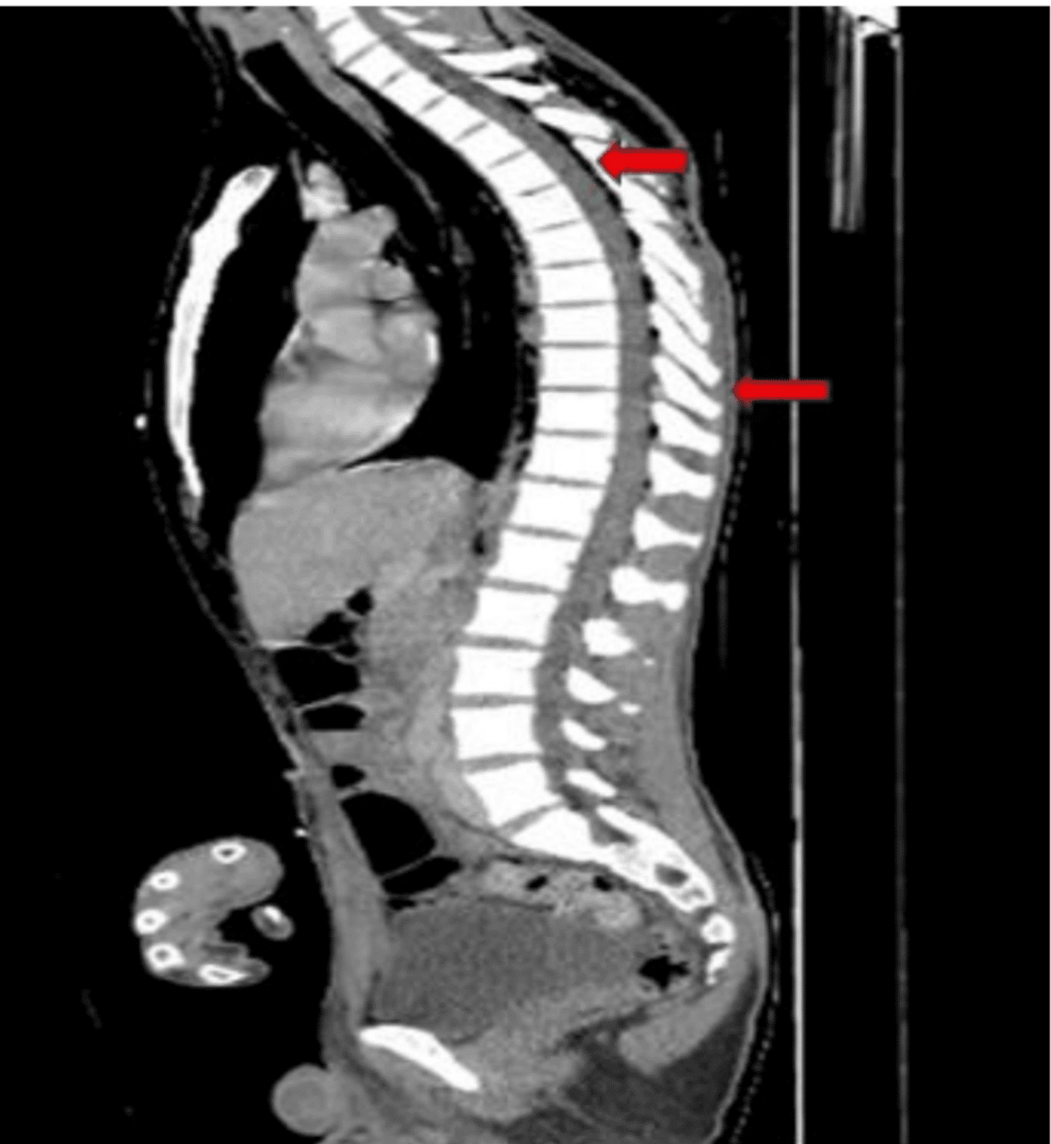
Computed tomography scan (sagittal view) showing dissection of air into the spinal cord (red arrows).

A CT combo body with intravenous (IV) contrast showed extensive pneumomediastinum (Figures 3, 4). Extensive soft tissue emphysema was noted with dissection of air into the spinal canal.

Given the patient’s imaging findings and a history of intractable vomiting, an etiology for his diffuse pneumomediastinum was likely Boerhaave’s syndrome with possible esophageal leak. Cardiothoracic surgery was consulted and recommended a Gastrografin study which was completed and returned negative for extraluminal contrast extravasation. Upon further inquiry, the patient admitted to taking fentanyl and Percocet from his friend the night he syncopated. During his admission, the patient developed severe bilateral upper and lower extremity weakness secondary to rhabdomyolysis with creatine kinase of 1,319 UL as well as sepsis secondary to methicillin-sensitive *Staphylococcus aureus* (MSSA) bacteremia as seen on blood culture results which were likely secondary to decubitus ulcer and wounds noted on his body.

The pneumomediastinum was managed conservatively, and the sepsis was treated with IV cefazolin 2 g every eight hours, IV metronidazole 500 mg twice daily, and IV micafungin 100 mg daily. After a 10-day hospital stay, the patient was discharged in stable condition with cefadroxil 1 g tablet every 12 hours for 12 days for his MSSA bacteremia.

## Discussion

Pneumomediastinum was first described by Laennec in 1819 and was believed to occur only due to traumatic injury [9]. It can be benign and resolve on its own, but it can also be life-threatening in certain situations, such as esophageal perforation or mediastinitis, requiring surgical intervention [9,10].

PSPM is less severe and results from non-traumatic causes that increase intrathoracic pressure, e.g., pulmonary bleb rupture, smoking, or drug use [11]. On the other hand, SPM is caused by trauma and iatrogenic injury from excessive coughing and vomiting [1]. Distinguishing between the two is vital as SPM has higher morbidity and mortality rates and requires a more extensive treatment and recovery period [12] than PSPM which is managed conservatively.

In SPM, direct trauma or injury to the esophagus, alveoli, or tracheobronchial tree results in air leakage along the peribronchial and perivascular sheaths into the mediastinum, a phenomenon known as the Macklin effect [13]. Increased tension in the closed cavity can compress the heart, lungs, and arteries, potentially compromising respiratory and vascular capability. In this case, the patient had been vomiting for 2.5 weeks and had chest pain for 3-4 days before seeking medical help. He had taken “street Percocet” laced with fentanyl to manage the chest pain. He also sustained trauma from multiple falls during a hunting competition. He was found improperly clothed and unconscious in 16°F weather and presented with nares, fingers, and toes frostbite. The combination of trauma, vomiting, drug use, and exposure to freezing temperatures led to the patient developing secondary pneumomediastinum concurrently with pneumorrhachis diagnosed radiographically.

Pneumorrhachis can occur in 5.8-9.5% of pneumomediastinum cases [14,15]. The absence of fascial barriers between the posterior mediastinum and the epidural/retropharyngeal spaces allows air to diffuse through the neural foramina into the epidural space [16,17]. While spinal epidural air is usually reabsorbed without neurological issues, intra-axial spinal air often indicates a serious cranial or spinal injury and should be medically investigated [18]. Behr et al. reviewed 242 instances of pneumomediastinum with 15 cases of concomitant pneumorrhachis. In all 15 cases, no neurological symptoms were present. No statistically significant relationship was reported between the increased severity of pneumomediastinum, the incidence of pneumorrhachis and PSPM compared to SPM, and the incidence of pneumorrhachis [14]. Pneumorrhachis was also discovered in patients with air distribution in all three mediastinal compartments more significantly than those with air distribution in fewer compartments. Additionally, Belotti et al. reviewed 48 cases of pneumorrhachis related to PSPM, and 98% of the cases revealed no neurological symptoms with spontaneous illness resolution [15]. However, in some cases, pneumorrhachis does cause neurological symptoms. Physical examination may reveal precordial crepitus or mediastinal “crunch/click” that synchronizes with the patient’s heartbeat, a phenomenon known as Hamman’s sign, in 30% of cases [19,20]. Furthermore, subcutaneous emphysema occurs in approximately 70% of pneumomediastinum cases [20].

Morgan et al. outlined the signs, symptoms, diagnostics, and treatments seen in 535 pneumomediastinum patients in 19 case series reviewed. The review found a 3:1 male-to-female ratio with an average mean age of 23 years. The most prevalent sign on physical examination was subcutaneous emphysema (54.2%) [21]. The most predominant symptom, present in 70.9% of the patients, was chest pain. The second and third most frequent symptoms, reported in 43.4% and 32% of the patients, respectively, were dyspnea and neck discomfort [21]. The common social and medical history reported were smoking (29.6%), coughing (27.7%), asthma (25.9%), physical exercise (21.1%), and recent emesis (13%) [21]. Almost all patients (96.9%) underwent chest X-rays. Overall, 65% underwent a CT scan, 35.6% an esophagogram (35.6%), 13% an esophagogastroduodenoscopy (13%), and 14.6% a bronchoscopy [21].

The patient in this case exhibited subcutaneous emphysema and crepitus over the right and left upper anterior chest wall, cervical spine, and upper/mid-thoracic spine. He underwent a chest X-ray, CT of the head without contrast, CT of the cervical spine, CT combo body, and esophagogram. CT body combo revealed extensive pneumomediastinum with extensive soft tissue emphysema and dissection into the cervical spinal canal, consistent with the physical examination findings. Esophagograms are crucial for ruling out esophageal perforation, a life-threatening cause of subcutaneous emphysema that must always be investigated in pneumomediastinum patients. In this case, the esophagogram did not show evidence of extraluminal contrast leakage.

Here, we presented the case of a 27-year-old male with a documented history of polysubstance abuse who presented with a two-day history of intractable emesis, nausea, and chest tightness. Advanced imaging techniques such as CT combo body scans and esophagograms aided in reaching a timely diagnosis of PSPM and non-traumatic pneumorrhachis. The need for a timely esophagogram study is crucial for ruling out esophageal perforation, a life-threatening cause of subcutaneous emphysema. With timely diagnosis, the average hospital stay is approximately four to five days, with an admission rate of 86.5%. However, the finding of MSSA bacteremia, electrolyte derangements, acute kidney injury, transaminitis, suspected intussusception, rhabdomyolysis, severe lumbar pain, pitting edema/paresthesia in the bilateral feet, and frostbite during the investigation prolonged the patient’s hospital stay to 10 days. He recovered fully and was discharged in a stable condition without any significant respiratory or neurological sequelae.

## Conclusions

Pneumomediastinum is a benign condition that is occasionally associated with emphysema and pneumothorax, but it is rarely associated with pneumorrhachis. A chest X-ray and a compatible clinical and physical examination are typically sufficient for diagnosis. CT scan is employed when chest X-rays are non-diagnostic. In a situation where intractable vomiting is reported, employing an esophagogram study to rule out esophageal perforation is highly recommended. This case demonstrates the importance of appropriate diagnosis in a patient presenting with multifactorial causes of pneumomediastinum and the importance of addressing possible life-threatening causes.
